# Highly active promoters and native secretion signals for protein production during extremely low growth rates in *Aspergillus niger*

**DOI:** 10.1186/s12934-016-0543-2

**Published:** 2016-08-20

**Authors:** Franziska Wanka, Mark Arentshorst, Timothy C. Cairns, Thomas Jørgensen, Arthur F. J. Ram, Vera Meyer

**Affiliations:** 1 Department Applied and Molecular Microbiology, Institute of Biotechnology, Berlin University of Technology, Gustav-Meyer-Allee 25, 13355 Berlin, Germany; 2 Department Molecular Microbiology and Biotechnology, Institute of Biology Leiden, Leiden University, Sylviusweg 72, 2333 BE Leiden, The Netherlands; 3 Protein Expression, Novo Nordisk, Novo Nordisk Park, 2760 Måløv, Denmark

**Keywords:** Perfusion cultivation, Zero growth rate, Antifungal protein, Hydrophobin, Promoter, *Aspergillus niger*

## Abstract

**Background:**

The filamentous ascomycete *Aspergillus niger* is used in many industrial processes for the production of enzymes and organic acids by batch and fed-batch cultivation. An alternative technique is continuous cultivation, which promises improved yield and optimized pipeline efficiency.

**Results:**

In this work, we have used perfusion (retentostat) cultivation to validate two promoters that are suitable for *A. niger* continuous cultivation of industrially relevant products. Firstly, promoters of genes encoding either an antifungal protein (*Panafp*) or putative hydrophobin (*PhfbD*) were confirmed as active throughout retentostat culture by assessing mRNA and protein levels using a luciferase (*mluc*) reporter system. This demonstrated the *anafp* promoter mediates a high but temporally variable expression profile, whereas the *hfbD* promoter mediates a semi-constant, moderate-to-high protein expression during retentostat culture. In order to assess whether these promoters were suitable to produce heterologous proteins during retentostat cultivation, the secreted antifungal protein (AFP) from *Aspergillus giganteus*, which has many potential biotechnological applications, was expressed in *A. niger* during retentostat cultivation. Additionally, this assay was used to concomitantly validate that native secretion signals encoded in *anafp* and *hfbD* genes can be harnessed for secretion of heterologous proteins. *Afp* mRNA and protein abundance were comparable to luciferase measurements throughout retentostat cultivation, validating the use of *Panafp* and *PhfbD* for perfusion cultivation. Finally, a gene encoding the highly commercially relevant thermal hysteresis protein (THP) was expressed in this system, which did not yield detectable protein.

**Conclusion:**

Both *hfbD* and *anafp* promoters are suitable for production of useful products in *A. niger* during perfusion cultivation. These findings provide a platform for further optimisations for high production of heterologous proteins with industrial relevance.

**Electronic supplementary material:**

The online version of this article (doi:10.1186/s12934-016-0543-2) contains supplementary material, which is available to authorized users.

## Background

An inherent component of the saprophytic lifestyle of the filamentous mould *Aspergillus niger* is the ability to secrete large amounts of enzymes into its environment, which has been harnessed in biotechnological pipelines for the efficient production of platform chemicals and industrial proteins. In recent years, improved morphological [1], genetic [2], metabolic [3], and systems biological tools [4, 5] offer improved efficiency and tractability of *A. niger* in industrial applications.

However, innovations specifically tailored to improving bioprocess strategies have been limited. Currently, approximately 90 % of industrial biotechnological cultivations rely on batch or fed-batch culture [6], which is often inefficient as organisms have short periods of high product biosynthesis, and there is considerable manufacture downtime for technical reasons, such as equipment sterilization. Additionally, fed-batch or batch cultivation can result in inconsistent product quality (e.g. multiple glycosylation variants) because of the disparities in medium environment [7].

An alternative and potentially useful strategy for biotechnological manufacture is continuous processing. Chemostats, in which fresh medium is continually added to a bioreactor, and effluent containing metabolite products, used medium, and microbial biomass continually removed, enables steady state microbial growth. Accordingly, optimal growth rates for product biosynthesis can be maintained, and the period of product biosynthesis increased when compared to batch cultivation [8].

A modification of conventional chemostat cultivation is termed perfusion or retentostat cultivation, in which microbial biomass is retained in the bioreactor. Consequently, in retentostat cultivation, microbial biomass increases to a maximum biomass, after which available nutrients are sufficient for maintenance of cell viability, and growth rates approach zero. Additionally, perfusion cultivation has several advantages to conventional steady state chemostat cultivation. Firstly, extremely low microbial growth may increase available metabolic energy for product biosynthesis, thus potentially improving product yield. Secondly, many microbial secondary metabolite products are only produced during phases of low or zero growth, and accordingly novel products or those previously recalcitrant to batch or fed-batch cultivation might be amenable to retentostat biosynthesis. Another advantage of this cell retention cultivation mode is the continuous removal of toxic or growth inhibitory products and/or the production of unstable products, which cannot remain stable in a batch or fed-batch culture due to inherent sensitivities to proteases or other degradative enzymes. Additionally, this kind of cultivation enables continuous product monitoring and prompt downstream processing of secreted metabolites or enzymes. A major advantage is the high productivity in small-scale bioreactors, which save money, space, and allow an easier scale up process. Accordingly, expanding the cultivation tool-kit of *A. niger* to include perfusion cultivation is an important goal in biotechnology.

Currently, a significant technical challenge to the development of efficient *A. niger* perfusion cultivation is the absence of suitable promoter systems. For example, conventionally used promoters for high expression in industrial systems (e.g. the glucoamylase promoter *PglaA*) show prohibitively low expression activity at growth rates which are close to zero [9]. Discovery of promoters with high activity at ultralow growth rates is a prerequisite for *A. niger* retentostat cultivation of useful products. Several other factors for optimal promoter functionality include activity in the absence of an inducer for simple recovery of desired product from culture medium, and continual promoter activity over a maximal time-period.

The objective of the study was to identify and validate *A. niger* promoters suitable for retentostat cultivation, and provide proof of principle for retentostat biosynthesis of heterologous proteins with potential biotechnological applications. Accordingly, from a previous transcriptomic analysis of *A. niger* retentostat culture [9], we rationally selected promoter regions of two genes, one encoding the *A. niger* antifungal protein (ANAFP; An07g01320), and the other encoding a putative hydrophobin (HFBD; An08g09880), both of which had high transcript abundance and strong supporting evidence of gene transcription during low *A. niger* growth [9]. Using a luciferase reporter for highly accurate readouts of promoter activity, we validated that both these promoters resulted in high heterologous gene transcription and protein translation during retentostat culture. In order to provide proof of principle that: (i) this platform can be used for cultivation of heterologous proteins with biotechnological applications, and (ii) native secretion signals encoded in these genes are useful for protein secretion during cultivation, both promoters and native secretion signals were used to express a gene encoding an *A. giganteus* antifungal protein, which has promising applications as a novel therapeutic in agriculture and the clinic. Lastly, a gene encoding the highly commercially relevant thermal hysteresis protein (THP) from *Choristoneura fumiferana* isoform 337 [10] was expressed during perfusion culture, which was unsuccessful for protein production, which we hypothesise is due to poor codon usage of this gene or proteolytic degradation.

## Results and discussion

### Establishment of zero growth active promoters in *A. niger*

We have previously interrogated global transcriptional changes of *A. niger* wild type strain N402 during retentostat cultivation, which identified a striking transcriptional increase for genes encoding putatively secreted, small molecular weight, cysteine-rich proteins relative to batch cultivation controls [9]. Accordingly, promoters from these genes were hypothesized to be good candidates for maintaining high expression of heterologous genes during retentostat cultivation. From this subset we rationally selected promoters upstream of: (i) a gene encoding the *A. niger* antifungal protein (An07g01320), which has been previously characterized as both expressed and translated during periods of low *A. niger* growth [9, 11], and (ii) a gene encoding a putative hydrophobin (An08g09880), for which expression levels from global transcriptional profiling were validated by Northern blot probe, thus giving high confidence of expression throughout retentostat culture [9].

**Fig. 1:**
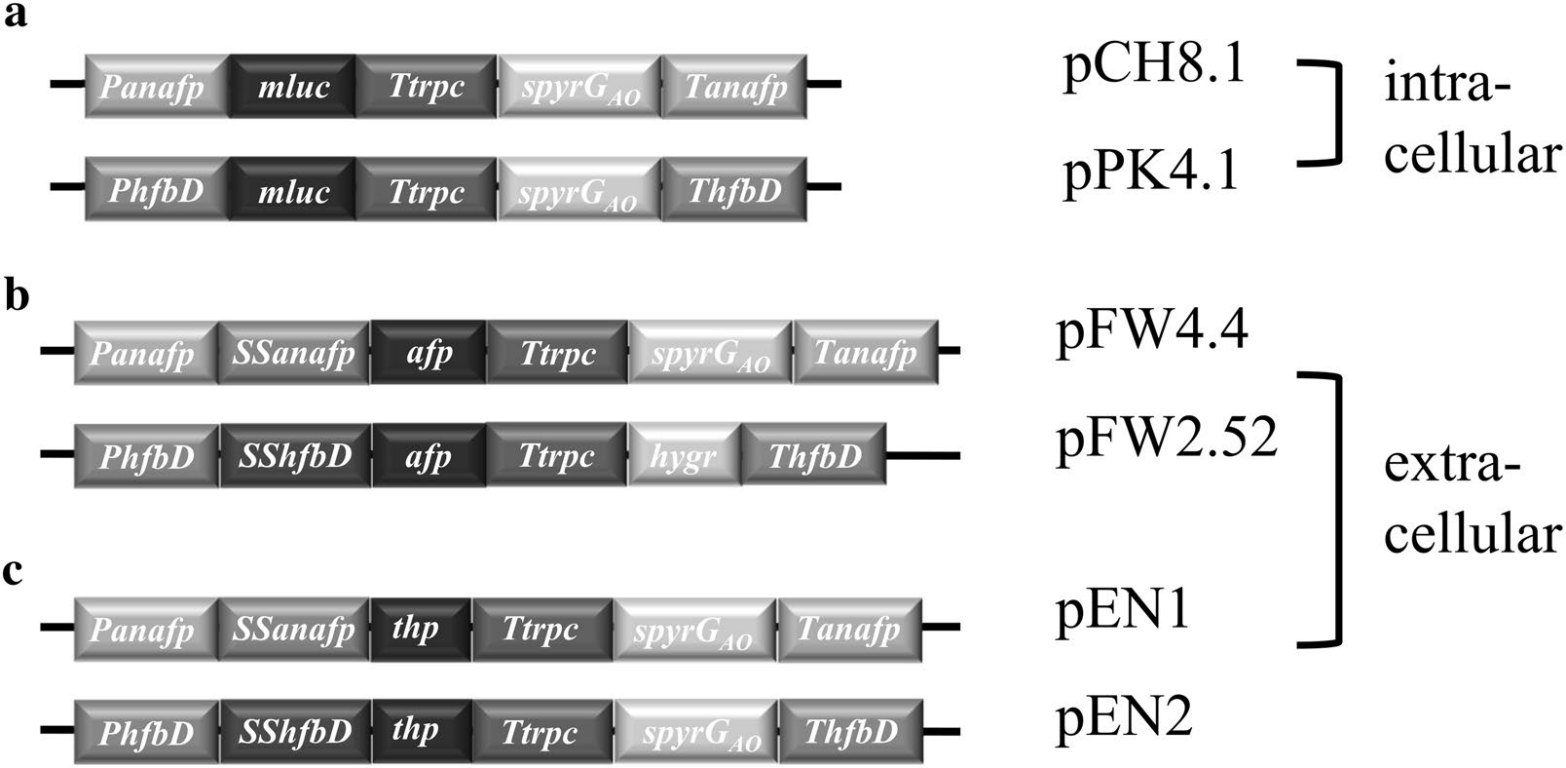
Schematic diagram depicting plasmids utilized to test activity of promoters during extremely low *A. niger* growth. For plasmids pCH8.1, pFW4.4 and pEN1, the promoter for the antifungal protein from *A. niger* (*Panafp*) and the corresponding terminator (*Tanafp*) were used. In the other three plasmids (pPK4.1, pFW2.52, pEN2) the promoter *PhfbD* and terminator *ThfbD* were utilized. These ~1000 bp promoter and terminator regions ensure homologous integration of exogenous DNA cassettes at the respective native locus via a double crossover event. In all plasmids, termination of transcription of the gene of interest was attained using the terminator of tryptophan synthase of *Aspergillus nidulans*, *Ttrpc*. All plasmids utilized the short version of *pyrG* (*spyrG*_*AO*_) for selection of transformants, with exception of pFW2.52, which encoded the hygromycin resistance gene (*hygR*). **a** For facile intracellular reporting of promoter activity, plasmids CH8.1 and PK4.1 encode the modified luciferase gene *mluc* [16]. **b** Plasmids FW4.4 and FW2.52 contain the *afp* gene encoding the *A. giganteus* antifungal protein and additionally the signal sequence for secretion of the respective genes (*SSanafp* and *SShfbD*). **c** For expression of the thermal hysteresis protein gene from the spruce budworm *C. fumiferana,* the codon optimized (for *A. niger*) *thp* gene was utilized in plasmids EN1 and EN2

To validate the functionality of the promoter regions for production of heterologous proteins, we generated DNA cassettes that replaced coding sequences for An08g09880 or An07g01320 at the native genomic locus with one of three genes (Fig. 1). DNA cassettes with the *mluc* gene encoding a luciferase protein were used for facile intracellular reporters of heterologous gene transcription and translation (Fig. 1a). Additionally, in order to demonstrate retentostat cultivation using these promoters could produce proteins of industrial relevance, AFP and THP encoding genes were also cloned into An07g01320 or An08g09880 loci. These DNA cassettes were also designed to validate whether native signal sequence of ANAFP or HFBD encoding genes can be harnessed for efficient secretion of heterologous proteins. Accordingly, DNA cassettes pFW4.4 (pEN1) and pFW2.52 (pEN2) contained codons encoding 34 and 22 amino acids of the predicted native secretion signal of An07g01320 and An08g09880 respectively at the N-terminus of the protein of interest (Fig. 1b, c).

In order to accurately assay biosynthesis of *A. giganteus* AFP at the *hfbD* locus, it was first necessary to delete the gene encoding *A. niger* AFP (An07g01320) as this polypeptide would likely be co-purified with heterologously expressed AFP (MA170.27, Table 1). This Δ*anafp* isolate was then used as a recipient strain for heterologous expression of *A. giganteus* AFP following transformation with pFW2.52, with this latter transformation utilizing a hygromycin gene as selection marker (Fig. 1b). All other plasmids utilized the short version of orotidine-5′-decarboxylase gene from *Aspergillus oryzae* (*spyrG*_*AO*_) as a selection marker. All strains used in this study are described in Table 1.

**Table 1:** *Aspergillus* strains used in this study

Strain	Genotype	Source
MDH18894	*A. giganteus* wild type	[12]
N402	*A. niger* wild type	[13]
AB4.1	*A. niger pyrG* ^−^ isolate	[14]
PK2.9	AB4.1 transformed with pCH8.1 (*Panafp*::*mluc*), *pyrG* ^+^	This work
PK4.3	AB4.1 transformed with pPK4.1 (*PhfbD*::*mluc*), *pyrG* ^+^	This work
FW23.7	AB4.1 transformed with pFW4.4 (*Panafp*:: *SSanafp*::*afp*), *pyrG* ^+^	This work
MA170.27	AB4.1 transformed with PCR product of pCH3.3(*Panafp*::*spyrG*_*AO*_::*Tanafp*) → Δ*anafp*, *pyrG* ^+^	This work
FW6.6	MA170.27 transformed with pFW2.52 (*PhfbD*::*SShfbD*::*afp*), *hygR* ^+^ *, pyrG* ^+^	This work
MA237	AB4.1 transformed with pEN1 (*Panafp*::*SSanafp*::*thp*), *pyrG* ^+^	This work
MA238	AB4.1 transformed with pEN2 (*PhfbD*::*SShfbD*::*thp*), *pyrG* ^+^	This work

### Retentostat cultivation

**Fig. 2:**
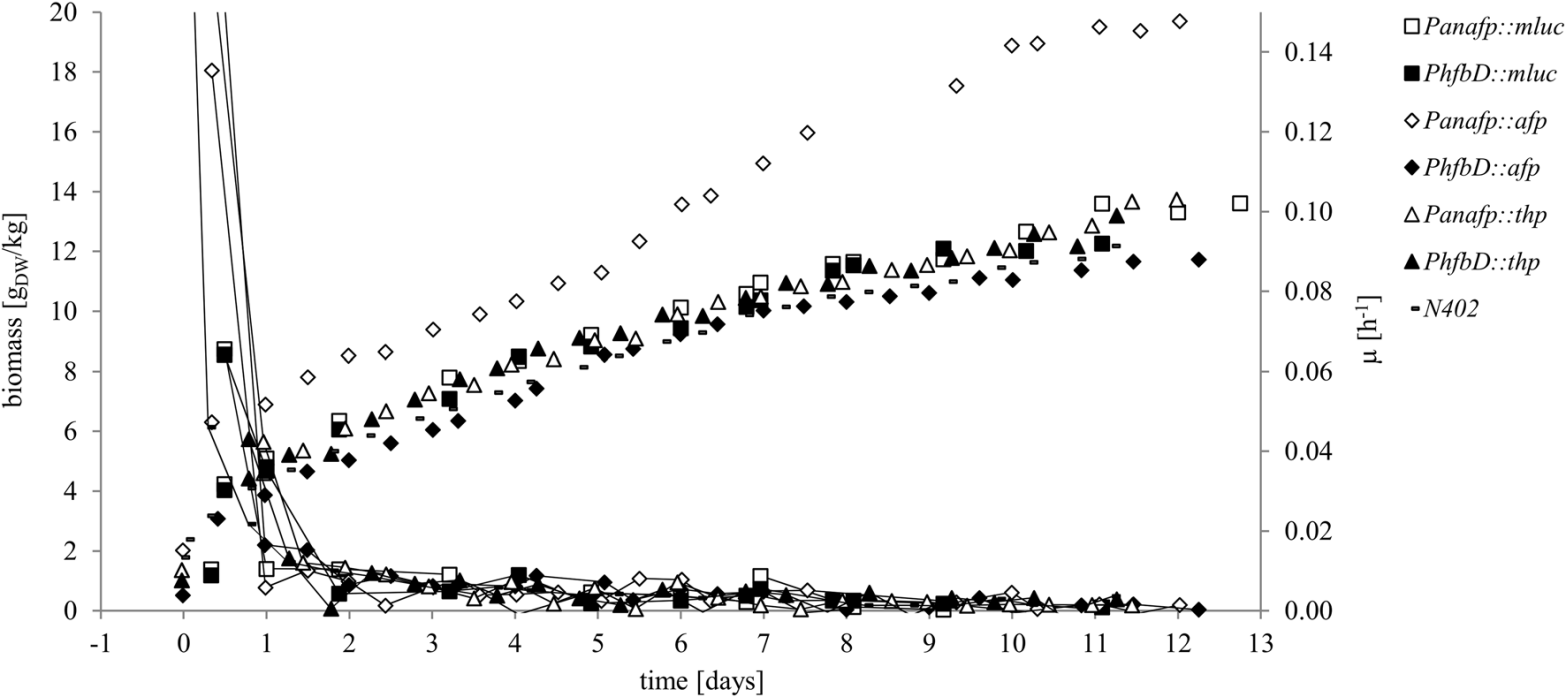
Biomass accumulation and specific growth rate. Biomass and specific growth rates (µ) are reported after a switch from batch to perfusion cultivation (day 0). Biomass values are reported as *unconnected points*, whereas growth rates are reported with* connecting lines*. The biomass concentration increased during the biomass retention from ~2.1 g_dry weight_/kg up to ~12 g_dry weight_/kg in 12 days. The strain FW23.7 (*Panafp*::*afp*) has the double amount of glucose available (batch: 0.8 % glucose, perfusion: 1.6 % glucose), resulting in an approximately ~6 h earlier switch to perfusion cultivation, and a final biomass after 12 days of ~20 g_dry weight_/kg. The *right* diagram axis shows strain specific growth rate, which decreased within 2 days of changing cultivation conditions and reached values close to zero. For each strain one single bioreactor run was performed. N402 data taken from [9]

*Aspergillus niger* isolates expressing DNA cassettes (Table 1) were cultivated under retentostat conditions for 13 days in 5 l bioreactors. These data were compared to wild type progenitor isolate N402 grown under perfusion cultivation conditions [9]. Biomass and specific growth rates (µ) were calculated throughout the time series of cultivation at 12 h intervals (Fig. 2). After 5 days (±12 h), specific growth rate of all fungal isolates approached zero (Ø = 0.004 h^−1^). Approximately at the same time point, the coloration of the culture was visibly darkened, which was verified to be due to melanized spore production (data not shown), a process associated with low growth and secondary metabolism in filamentous fungi [9]. Melanin production of expression strains MA237 and MA238 was indistinguishable from the published data of wild type *A. niger* isolate N402 [9] (data not shown). The values of extremely low specific growth rates approached zero (lowest estimated value 0.00029 h^−1^, day 12, strain FW6.6) within 13 days cultivation. These data are comparable with specific growth rates of *Saccharomyces cerevisiae* retentostat cultivations, which reach 0.0006 ± 0.0001 h^−1^ after 22 days [15]. The perfusion cultivations of PK strains were fermented with maltose as carbon source, whereas the cheaper carbon source glucose was used for MA and FW strains. This resulted in comparable amounts of biomass between samples. In order to test if an increase in biomass would improve the titre of heterologous protein expression during perfusion cultivation, we amended growth media for strain FW23.7 to double the glucose concentration, which resulted in a clear increase in biomass, but no significant change in absolute growth rate. Moreover, specific growth rate and AFP protein production (Figs. 2, 4) of this strain was only slightly impacted by glucose availability and biomass, thus providing evidence of decoupling *A. niger* biomass formation from protein production. Accordingly, retentostat cultivation is a robust and titratable platform for *A. niger* heterologous protein production.

### Analysis of mRNA and luminescence levels of a luciferase reporter demonstrate high activity of *anafp* and *hfbD* promoters during perfusion cultivation

**Fig. 3:**
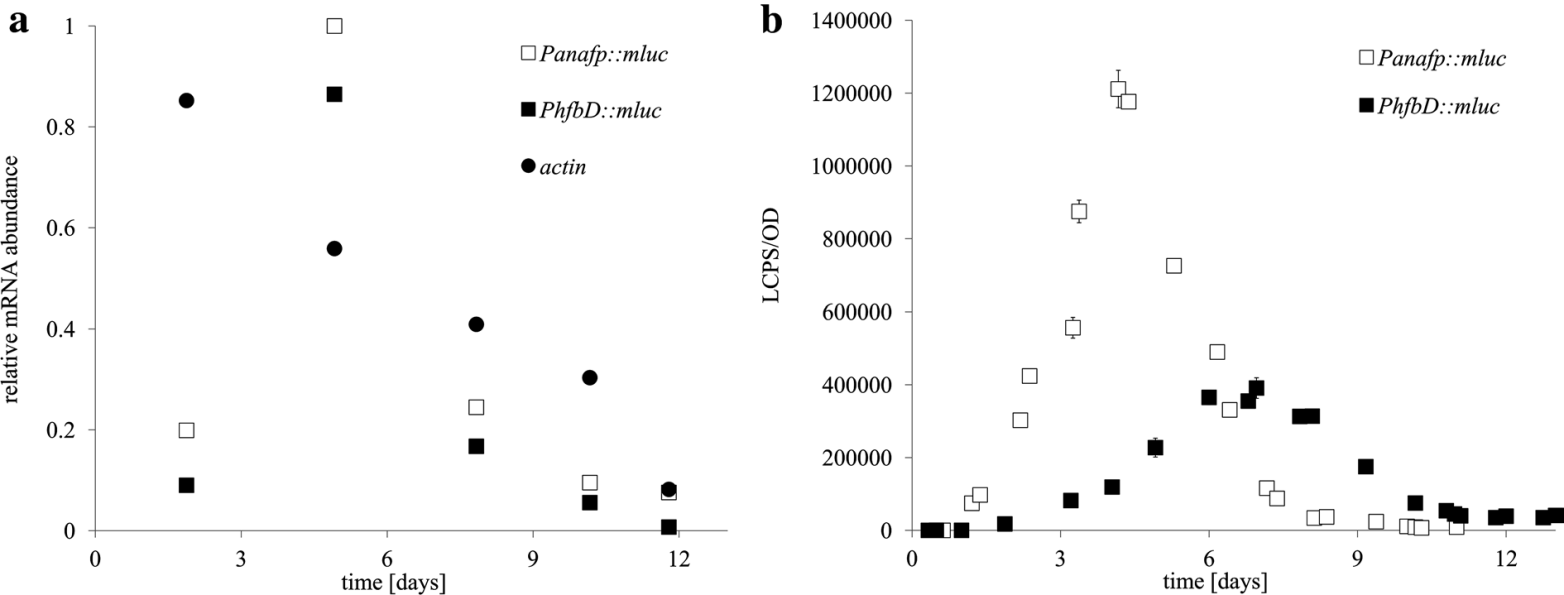
*mluc* mRNA and protein expression analysis throughout a time-course of perfusion cultivation. **a** Quantification of mRNA abundance from Northern blot analyses with the *mluc* probe demonstrated gene expression directed by the *anafp* promoter in strain PK2.9 was highest following 5 days perfusion cultivation, which was also the case for promoter *PhfbD* in strain PK4.3. As a control, mRNA abundance for the housekeeping gene actin was measured in strain *PhfbD*::*mluc*, which demonstrated a decrease in relative transcript abundance over time (*closed circles*). **b** Protein abundance was measured as luminescent counts per second normalized to culture optical density, which clearly demonstrated highest protein concentration in strain PK2.9 using the *anafp* promoter when compared to isolate PK4.3 (*PhfbD*)

**Fig. 4:**
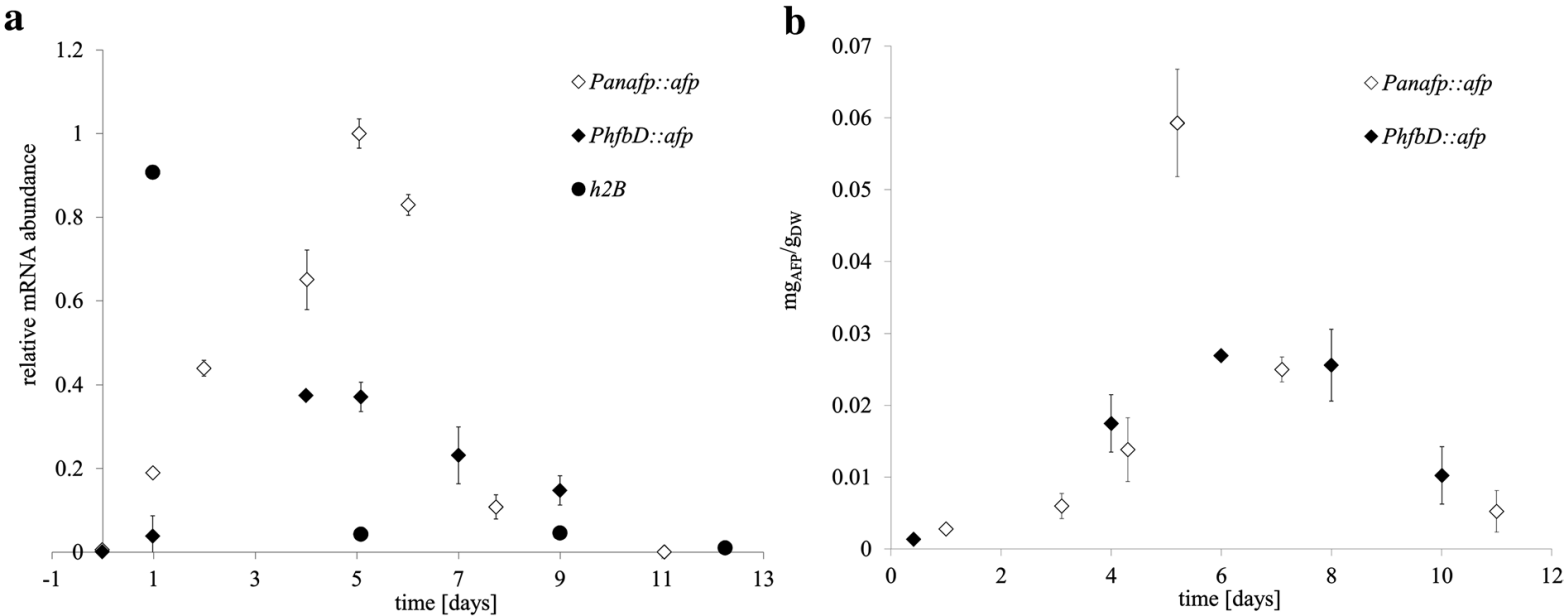
*afp* mRNA and protein expression analysis throughout a time-course of perfusion cultivation. **a** *afp* mRNA levels, pictured in *diamonds*, were measured using qRT-PCR throughout a time period of perfusion cultivation from strain FW23.7 (*Panafp*::*afp*) and FW6.6 (*PhfbD*::*afp*). Reported standard deviation resulted from a minimum of two and maximum of three measurements per sample. These data support temporal expression profiles reported for *mluc* gene, with *afp* expression highest at day 5 for both *Panafp* and *PhfbD*. As a control the housekeeping gene *h2B* was measured in strain *PhfbD*::*afp*, which demonstrated a decrease in relative transcript abundance over time (*closed circles*). **b** Protein abundance was quantified using Western blot (anti-AFP primary antibody) which demonstrated that *Panafp* produced maximal 0.06 mg_AFP_/g_dry weight_ corresponding to 0.65 mg_AFP_/l. Standard deviation was estimated through three Western blots per sample

**Fig. 5:**
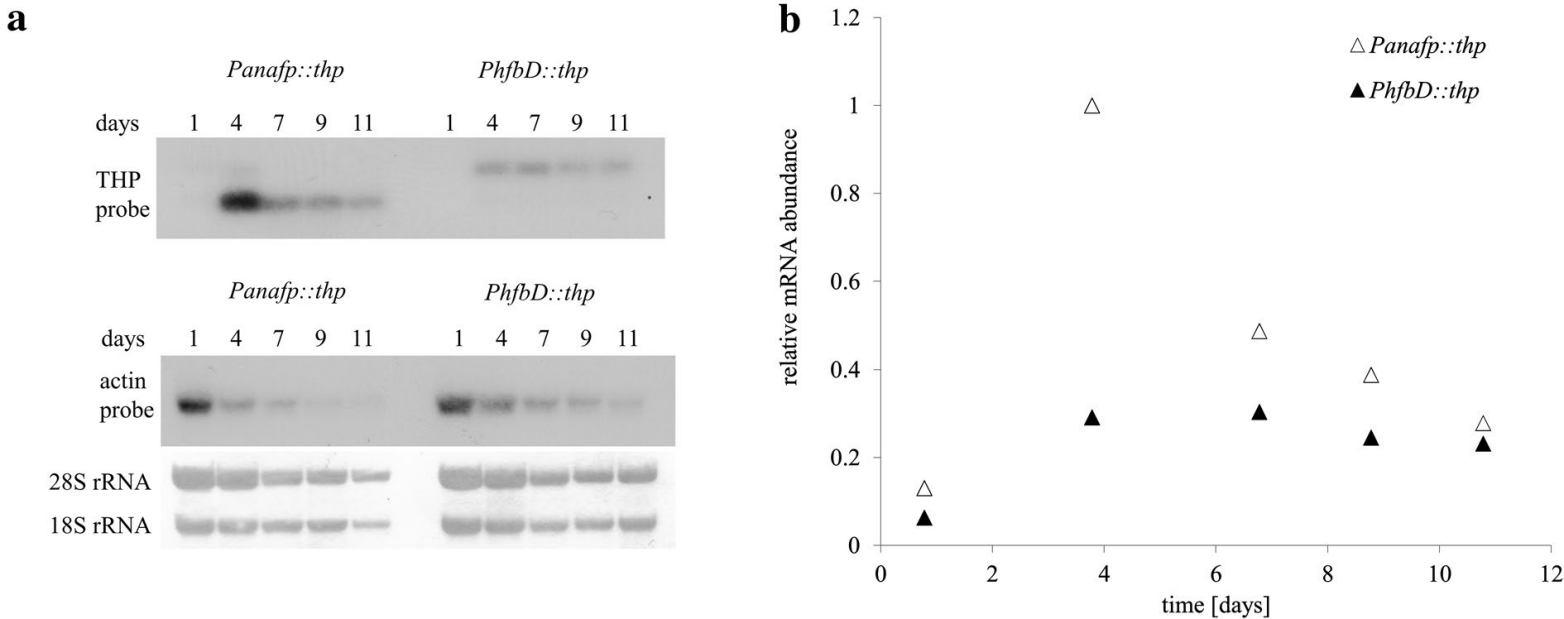
*thp* mRNA analysis throughout a time-course of perfusion cultivation. **a** Northern blot analysis of mRNA extracted from strains MA237 (*Panafp*::*thp*) and MA238 (*PhfbD*::*thp*) during a time-course of perfusion fermentation are shown, with probes for THP mRNA (*upper panel*) and actin mRNA (*middle panel*). These data demonstrate *thp* mRNA is expressed in this system using both *anafp* and *hfbD* promoters. The* lower panel* demonstrates representative decrease of 18S and 28S rRNA on Northern blot membranes throughout the experiment time course, which we hypothesize is due to decrease in cellular metabolism. **b** Quantification of mRNA abundance from Northern blot results of *thp* (**a**) in strain MA237 (*Panafp*::*thp*) and strain MA238 (*PhfbD*::*thp*). These data suggest highest mRNA expression after 4 days of perfusion cultivation in isolate MA237, and approximately constitutive expression between days 4 until day 12 in isolate MA238

For comprehensive analysis of *anafp* and *hfbD* promoter activity throughout retentostat cultivation, we measured both mRNA and protein abundance for an intracellular reporter gene, *mluc* [16], using Northern blot probes and luciferase activity, respectively. This experiment highlighted a general and significant technical challenge for estimating mRNA transcript abundance during ultralow fungal growth, i.e. the absence of constitutively active housekeeping genes for normalization of mRNA levels for genes of interest. During retentostat culture, transcripts from actin, histone 2B (H2B), 18S and 28S ribosomal encoding genes were tested using Northern blot probes, which demonstrated a clear reduction in transcript abundance throughout the experiment as exemplarily shown in Figs. 3a, 4a and 5, an observation supported by global transcriptional profiling as previously reported for *A. niger* perfusion cultivations [9]. Appropriate reference genes for mRNA normalization for industrially important fungi is highly complex and dependent on the experimental context [17]. Accordingly, we report mRNA abundance at each time point normalized to the highest measurement throughout the experiment, reasoning that this will enable interpretation of relative temporal changes of mRNA abundance throughout this experiment.

mRNA levels demonstrated that *mluc* transcripts were highest for both *anafp* and *hfbD* promoters at day 4 (Fig. 3a), at which time the specific growth rates of strains PK2.9 and PK4.3 were approaching zero (0.007 h^−1^, respectively). Encouragingly, this suggested that decoupling biomass accumulation and heterologous protein expression had been achieved in this system.

This was broadly supported by analysis of protein expression, which for strain PK2.9 P*anafp* showed a peak in luciferase activity at day 4 [1,200,000 luminescent counts per second (LCPS)/optical density (OD)] which dropped to relatively lower, but still detectable luciferase values (34,000 LCPS/OD) by day 8 (Fig. 3b). In strain PK4.3 with *PhfbD*, luciferase values were highest at day 7 (400,000 LCPS/OD), and were detectable at 40,000 LCPS/OD by day 11 (Fig. 3b). While these data demonstrate promoters *anafp* and *hfbD* are not constitutively active throughout retentostat culture, greater LCPS/OD values were achieved in this system than during conventional batch cultivation of *A. niger* strain expressing *mluc* under control of a titratable, tetracycline inducible expression system (at 41 h, 250,000 LCPS/OD) [18]. These data indicate promisingly high levels of protein expression using *Panafp* and *PhfbD* as promoters. Additionally, the duration of *anafp* and *hfbD* promoter activity is longer than in a batch culture (+5 days, respectively), indicating a potential improvement in duration of heterologous product expression in this pilot experiment.

### Proof of principle that perfusion cultivation using ultralow growth promoters can be used for heterologous expression of industrially relevant products

In order to provide proof of principle that perfusion cultivation using *anafp* and *hfbD* promoters enables expression of industrially relevant products, we generated strains FW23.7 and FW6.6 expressing a gene encoding the *Aspergillus giganteus* antifungal protein (Table 1). *Aspergillus giganteus* AFP is a small and compact molecule, containing four disulfide bridges and other tertiary noncovalent connectivities, which consequently is highly resistant to protease degradation [19]. The activity of antifungal protein is restricted to moulds with no cytotoxic effects detected on yeast, bacteria, plant and mammalians cells [20], which enables a wide-range of conceivable applications. The heterologous production of AFP failed so far in *E. coli* (own unpublished data), yet was successful in *P. pastoris* with 2.5 mg/l of mature protein [21] and in *A. niger* but with only low titres (350 µg/l [12]). *Aspergillus niger* itself is very sensitive against AFP with a MIC (minimal inhibitory concentration) of ~1 µg/ml (complete inhibition of germination) and ~80 µg/ml for the fungicidal effect on hyphae [22]. Therefore, we assume that the retentostat mode is a useful cultivation, due to the high dilution of the secreted toxic products, and the fact that the chosen promoters are not active during germination or exponential growth phase.

In order to confirm that mRNA decrease of housekeeping genes over the period of zero growth conditions was not due to an artefact of Northern blot analysis, we measured mRNA transcript abundance of *h2B* (Fig. 4a) and *gpdA* (data not shown) genes with qRT-PCR, which confirmed mRNA transcript of these genes decreased throughout perfusion fermentation. mRNA measurements of *afp* transcripts in FW23.7 (*Panafp::afp*) and FW6.6 (*PhfbD::afp*) using qRT-PCR revealed comparable temporal expression patterns to *mluc*, indicating promoter activity was consistent between the different expression cassettes (Fig. 4a). In effluent extracted throughout perfusion cultivation, AFP protein was detectable using an anti-AFP antibody at a maximum of 0.065 mg_AFP_/g_DW_ (using promoter *anafp*) and 0.03 mg_AFP_/g_DW_ (using promoter *hfbD*) estimated in comparison to the positive control of purified *A. giganteus* AFP (see “Methods” section). The calculated highest AFP production rate of FW23.7 (*Panafp*) was 0.8 mg/l/day at day 5 (µ = 0.004 h^−1^), and for FW6.6 (*PhfbD*) it was 0.08 mg/l/day from day 3 until 4, which corresponds to µ = 0.006 h^−1^. These differences may be due to improved functionality of the *Panafp* promoter, increased production of AFP under higher glucose concentrations used in growth media for strain FW23.7, or a combination of these factors.

In order to validate that heterologous expression of AFP using *A. niger* resulted in protein with potential biotechnological applications, heterologously produced AFP from strain FW6.6 (day 9) was purified via FPLC and tested for antifungal activity. 94 % growth inhibition of *A. niger* was achieved using 10 µg/ml heterologously expressed AFP, which is comparable to AFP purified from *A. giganteus* (100 % growth inhibition using 10 µg/ml) [22]. These data demonstrate that heterologously produced AFP is biologically active. The AFP yield using *A. niger* perfusion cultivation was 650 µg/l (FW23.7, day 5), which is a notable improvement when compared to previous AFP production using *A. niger* in shake flask culture (~350 µg/l) [12]. While shake flask production is not suitable for industrial applications, these data suggest that further optimization of perfusion fermentation is warranted. Elsewhere, groups using *P. pastoris* for AFP production have achieved yields of 2.5 mg/l [21]. However, all heterologous expression attempts are low in comparison to the homologous production host *A. giganteus* (30–40 mg/l), indicating further optimisation of all current systems is required for commerical use of AFP. Nevertheless, the significantly increased AFP yield reported here when compared to conventional purifications demonstrates perfusion cultivation is a promising technique which warrants future optimisation.

Taken together, these data are the first example of heterologous protein expression during perfusion cultivation using a filamentous fungal cell factory, and validate that promoters *anafp* and *hfbD* are sufficiently active for heterologous gene expression during ultralow growth. In *A. niger*, refinement and expansion of the promoter tool-kit has been an ongoing effort for nearly 30 years, with over 15 constitutive or inducible systems described [23]. Accordingly, discovery of two promoters that are functional for protein production during *A. niger* perfusion cultivation is an important step which will facilitate future optimization of this technique. With regards to use of native secretion signals *anafp* and *hfbD*, *A. niger* is a useful cultivation system due to its ability to secrete post-translationally processed, active recombinant proteins into cultivation media at high concentrations. Protein signal peptides are therefore of critical importance as they enable translocation of useful enzymes through cellular secretory machinery. Our data demonstrate that the *anafp* and *hfbD* signal sequences encoded in pFW4.4 and pFW2.52 enable secretion of heterologous protein during ultralow growth in perfusion cultivation.

### Perfusion cultivation of THP protein from *Choristoneura fumiferana*

In order to test whether non *Aspergillus* spp. derived proteins with existing technological applications could be heterologously expressed using the perfusion cultivation platform, we generated strains expressing the THP protein from *Choristoneura fumiferana* isoform 337 [10]. This protein inhibits growth and recrystallisation of ice, lowers freezing temperature, increases melting temperature, is used in the food industry to improve storage of frozen products, and has potential applications in tissue preservation for organ transplants [24]. Figure 5 depicts relative mRNA abundance as measured by Northern blot analysis of strain MA237 (*Panafp*::*thp*) and MA238 (*PhfbD*::*thp*) with *thp* probes, which show comparable temporal expression profiles as those described for *mluc* and *afp* genes.

However, throughout perfusion cultivation, THP was not detected in the effluent. The enrichment through a cooling finger [25] suggested that no active THP was secreted in the medium, as the ice layer did not show the expected cloudiness from previously reported ice-THP interactions, and further analysis in SDS-PAGE did not demonstrate protein bands of predicted molecular mass (data not shown). To exclude the possibility of THP remaining intracellular, proteins were extracted from mycelium and protein samples analysed by SDS-PAGE which did not reproducibly result in a band of expected molecular mass. Where putative bands were detected, samples were further analysed by HPLC–ESI–MS, but these could not be confirmed as THP. Western blots with the mycelium extracts using rabbit anti-THP antiserum [26] demonstrated a non-specific band of high molecular mass (data not shown). We speculate that the THP protein, which is natively expressed in lymph, is not suited for an extracellular expression in *A. niger,* or alternatively, the secreted THP degraded through instability problems due to pH or protease sensitivity. It is possible that further optimizations of the strain such as the use of protease-negative isolates [27], or modification of medium pH, may result in detectable THP protein, which could be tested in future experiments but is outside of the objectives of this work. We therefore conclude that the codon-optimized THP is not amenable to extracellular expression in *A. niger*.

### Comparison of both promoters and future optimisations

In general, measurements of mRNA and protein abundance throughout this study demonstrate that gene expression using the antifungal protein promoter throughout perfusion fermentation results in higher mRNA and translated protein at low growth rates (µ = 0.008–0.003 h^−1^) when compared to use of the hydrophobin promoter. While strains expressing genes under control of the antifungal promoter were highest at day 5, values approach zero at the later time periods, and gene expression is down-regulated to levels observed during batch fermentation. In contrast, the hydrophobin promoter results in a lower, yet semi-constitutive expression profile, with mRNA and protein abundance for *mluc* and *afp* genes remaining approximately constant between days 4 and 10 (µ = 0.007–0.001 h^−1^).

Analyses of protein abundance for *mluc* and *afp* genes expressed using both *hfbD* and *afp* promoters suggests that ultralow *A. niger* growth achieved following ~10 days retentostat culture results in reduced protein concentration, which we hypothesize is due to a reduction of the energy supply necessary for protein translation and processing. Consequently, for optimal protein biosynthesis, it may be necessary to keep absolute growth rates above a certain threshold, for example µ > 0.001 h^−1^ for strains using *PhfbD* to drive gene expression. This is supported by zero growth studies in multiple microorganisms, which suggests that calorie-restricted conditions lead to down-regulation of genes involved in protein synthesis and scarcity of ATP [28]. We conclude therefore that efficient production of biotechnology products during zero growth rates may not be possible. One possibility for future optimization would be to work with a promoter specific feeding rate to enable a constant glucose concentration/growth rate in the bioreactor, which may result in more efficient protein expression. Another potential improvement to this protocol would the use of non-sporulating *A. niger*, e.g. the deletion mutant *flbA* or *brlA* [29], to maximise available energy for protein synthesis, because produced biomass after day 4/5 consists of around 20 % melanin [9], and the process of sporulation itself has been shown to inhibit protein secretion [29].

## Conclusions

This study demonstrates that *A. niger* is suited for perfusion cultivation of heterologous proteins, and describes an experimental platform for future optimization studies of this technique. Using the well-established intracellular luciferase (*mluc*) system, promoters of two genes were validated as highly active during carbon-starvation mode of chemostat culture. Heterologous expression of the industrially relevant AFP provided the first proof of principle that perfusion cultivation at ultralow growth is possible in *A. niger.* Moreover, it validated that secretion signals of proteins ANAFP (An07g01320) and HFBD (An08g09880) enable efficient secretion of heterologous proteins into the culture media, a prerequisite for successful perfusion cultivation. The advancement of more efficient cultivation methods and associated molecular tools is an important step towards future utilization of fungal continuous expression systems in industrial biotechnology.

## Methods

### Construction of the plasmids and cloning

Plasmid design for *A. niger* transformation followed a strategy whereby ~1000 bp of 5′ and 3′ flanking regions of genes (*anafp* An07g01320, *hfbD* An08g09880) facilitated gene replacement by a double cross over event in the recipient strain AB4.1 or MA170.27 (plasmids maps made available on request). To analyse the expression profile of the two promoters, *mluc* [30] was chosen as an intracellular reporter gene, with a *trpC* terminator followed in the 3′ orientation by the short version of *pyrG* from *Aspergillus oryzae* as a selection marker (Fig. 1). Further constructs for extracellular expression using *anafp* contain additional signal sequences for secretion (presequence) and prosequence, whereas the *hfbD* gene lacks a prosequence and accordingly only encodes a presequence. In the case of *anafp*, the signal sequence, termed *SSanafp,* is encoded by 102 bp of DNA [20]. For efficient secretion, a codon encoding a leucine amino acid was introduced immediately after the signal peptide, which is absent in exogenous *A. giganteus* AFP but consistent with the initial amino acid in mature *A. niger* AFP [31]. The signal sequence for gene *hfbD*, termed *SShfbD* consists of a 66 bp DNA region [32], which for maximal secretion encodes an additional serine as the first amino acid of heterologously expressed proteins. A gene encoding the thermal hysteresis protein (THP) from *Choristoneura fumiferana* isoform 337 (AF263009) [33] was codon optimised for gene expression in *A. niger* and obtained from GeneArt™, Germany. The *afp* encoding gene from *A. giganteus* MDH 18894 was amplified with PCR from genomic DNA and cloned immediately downstream of leucine and serine codons in *anafp* or *hfbD* regions, respectively. All cloning procedures utilised conventional endonuclease digestion and ligation, after which reactions were transformed into competent *Escherichia coli* strain TOP10. DNA constructs were verified through restriction endonuclease analysis and sequencing.

### *A. niger* transformation, strains and molecular techniques

The transformation of *A. niger* isolates AB4.1 and MA170.27 and subsequent selection procedures for *pyrG*^+^ or *hygR*^+^ transformants respectively were performed using recently described protocols [34]. Fungal chromosomal DNA isolation was extracted from transformants following two rounds of selection purification, and positive transformants were confirmed with diagnostic PCR and Southern Blot analysis for single integration of the plasmids in the recipient strain. Depending on integration locus of the plasmid, DIG labelled DNA probes were used in the Southern blot, which have a homologous sequence in *Panafp* (5′:AGTACGACGAACTGCCGATA, 3′:AGTCGCTGAGATGTCGTTCA, product size: 510 bp) or in *PhfbD* (5′:GAGGCTGTGTATTTGGCGAG, 3′:CCTCTCATTACAGGCGGGAT, product size 498 bp). To confirm the absence of ectopic integration of DNA constructs in the genome, at least two different restriction enzymes were used in independent Southern blots. The *A. niger* strains used in this study are shown in Table 1. MA170.27 carried a deletion of the *anafp* gene, introduced through a double crossover with a PCR product of pCH3.3 (*Panafp*::*spyrG*_*AO*_::*Tanafp*) (5′:TTCCCCTGCTCCTTAGGCAG, 3′:AATTTCGACTTGGTGGTTAG, product size: 4.053 kbp). Strains were routinely cultivated on minimal medium (MM) plates containing 1 × ASP + N (50 x ASP + N: 3.5 M NaNO_3_, 550 mM KH_2_PO_4_, 350 mM KCl, pH 5.5), 2 mM MgSO_4_, 1× trace elements solution (modified from composition given by Vishniac and Santer [35], consisting of 1000x trace elements solution [10 g of EDTA, 4.4 g of ZnSO_4_·7H_2_O, 1.01 g of MnCl_2_·4H_2_O, 0.32 g of CoCl_2_·6H_2_O, 0.315 g of CuSO_4_·5H_2_O, 0.22 g of (NH_4_)_6_Mo_7_O_24_·4H_2_O, 1.47 g of CaCl_2_·2H_2_O, and 1 g of FeSO_4_·7H_2_O)], 1.5 % agar, and 1 % glucose. Alternatively, complete medium (CM) plates were used for routine culture, consisting of MM supplemented with 1 % yeast extract and 0.5 % casamino acids.

### RNA extraction

For RNA extractions from frozen bioreactor mycelium, samples were flash frozen in liquid nitrogen and ground using a pestle and mortar. The RNA was extracted with TRIzol^®^ reagent (Invitrogen) and analysed for quality in a spectrophotometer at an absorbance of 260 nm. For northern blot analyses, 5 µg of RNA was used for glyoxal RNA gel electrophoresis and after capillary blotting by means of radioactive hybridization probes the expression of *mluc*, *thp*, *actA* (An15g00560), and *h2B* (An11g11310). ^32^P-labeled primers used for the generation of the probes can be found in the Additional file 1. For visualization and relative quantification of northern blots, the image processing program (ImageJ) was used. A semi-quantitative calculation of relative mRNA abundance was used. Firstly, the highest probe intensity observed throughout the experiment was calculated. Next, this value was used to normalize all other probe intensities, by dividing all probe intensities by the highest value. For the transcription level investigation of the *afp* expressing strains, qRT-PCR was used. RNA was extracted as describe above and afterwards the quality was also checked by glyoxal RNA gel electrophoresis. High quality RNA with a ratio of 260/280 nm of 2, estimated with the spectrometer, was used for further analysis. Ten microgram RNA per time point were treated with DNAse (DNA-*free™* Kit Applied Biosystems). The amount of RNA was estimated with an 1-Step Kit (qPCRBIO SYGreen, PCR Biosystems), whereby cDNA synthesis takes place directly followed by quantification with SYBR Green PCR in the Stratagene Mx3005P cycler via ct (cycle threshold) value calculations [36]. Primers are listed in Additional file 1. For normalization of relative mRNA abundance, all ct values throughout the experiment were subtracted by the lowest ct value observed (corresponding to the mRNA transcript for P*anafp::afp* at day 5), which provided values of mRNA abundance relative to this time point.

### Perfusion cultivation cultures

Bioreactors BioFlo3000 (New Brunswick Scientific, NJ) with a working volume of 5 l were inoculated with 5 × 10^9^*A. niger* spores. For preparation of inoculums fresh conidia were harvested from CM plates in sterile sodium chloride solution (0.05 % Tween 80, 0.9 % NaCl) to avoid spore aggregation, which was filtered through sterile Miracloth (CalBiochem). Twenty litre reactor medium consists of 90 g NH_4_Cl, 30 g KH_2_PO_4_, 10 g KCl, 10 g MgSO_4_·7H_2_O, 20 ml modified trace metal solution at pH 3. As carbon source for the batch phase, 1 l of 80 g/l maltose was added for the cultivation of the *mluc* expressing strains (PK2.9, PK4.3), or glucose for all strains with extracellular expressed reporter genes. For retentostat phase 0.01 % polypropylene glycol and 1 l of 160 g/l respective C-source were added to 20 l bottles for each run. The cultivation of FW23.7 contained double the amount of glucose in batch and retentostat phase. During the cultivation a temperature of 30 °C was supported through a temperature sensor in combination with heat exchanger. In addition, cooling tubing was fixed around the reactor above the working volume to avoid fungal growth in the head plate. A pH of 3 was maintained with a glass electrode (405-DPAS, Mettler Toledo) in connection with computer controlled addition of 2 M NaOH. Sterile air was added with a gas flow of 1 l/min through a sparger, with an optical oxygen sensor (InPro^®^6000, Mettler Toledo) which monitored measured oxygen saturation to a level of at least 71 %. In the bioreactor run with double glucose concentration, minimum 54 % dissolved oxygen was maintained.

Conidial germination occurred 6 h after inoculation, which was enabled by addition of 0.003 % yeast extract in reactor media. To prevent the dispersal of the hydrophobic spores in the bioreactor headspace, the stirrer speed was limited to 250 rpm and air supplementation limited to the bioreactor head space. After 6 h, 0.01 % polypropylene glycol was added to bioreactor and rpm increased to 750. The air flow was disconnected from the headspace to aerate the culture through the ring sparger. The batch phase was changed to continuous cultivation after a consumption of 24 ml 2 M NaOH at a concentration of approximately 2.1 g_dry weight_ kg^−1^ biomass according to Iversen et al. [37]. The retentostat cultivation mode commenced with a feeding flowrate of 0.125 l/h (correspond to a dilution rate of 0.025 h^−1^), and installation of a special cell retention device which pumped out used medium [9]. Stable culture mass was achieved by monitoring retentostat culture by weight using an analytical balance. Broth samples for biomass, RNA or MLUC protein analyses were taken at 12 h intervals, with a maximum extraction of 100 g culture broth in 24 h in order to minimize impact on the cultivation. The sample preparation for determination of biomass concentration and RNA analysis was conducted as described in Jørgensen et al. [9]. For analysis of extracellular AFP or THP protein producing strains, 1.5 l of effluent was collected from the retention device every 12 h.

### Reporter protein analysis

Broth samples from the cultivation with strains PK2.9 and PK4.3 were used for the determination of promoter activity using the luciferase reporter protein. Bioluminescence measurements were made in triplicate, with 130 µl of sample and 70 µl luciferin substrate mix (1.4 mM luciferin) in 96-well microtiter plates. The luminescent counts per second (LCPS) at 537 nm and the optical density (OD) at 595 nm were measured using a Victor3™ Perkin Elmer reader. Melanin extraction was done with hot NaOH and measured at 425 nm as described previously [9, 38].

For determination of AFP protein concentration, the effluent was concentrated from 100 ml with Centrifugal Filter Units (with cut off above 3 kDa, Amicon^®^Ultra) and eluted in a volume of ~100 µl, after which samples were loaded on 16 % Tricine-SDS-PAGE gels and analysed via western blot (anti-AFP primary antibody, IPK Gatersleben), which were calibrated using a positive control of purified AFP from *A. giganteus* [22]. To evaluate heterologous AFP for antifungal bioactivity, a MIC (minimal inhibitory concentration) assay in microtiter plates was conducted, in which 100 µl 2xYPG medium (0.6 % yeast extract, 2 % bacto peptone, 4 % glucose, pH 4.5), 10 µl of 10^5^ spores/ml *A. niger* N402 and 90 µl purified 10 µg/ml AFP in FPLC buffer (0.05 M NaAc, 0.1 M NaCl) were combined [39]. For every condition three replicates were prepared and growth rates determined using OD measurements at 600 nm after 48 h incubation at 30 °C, and the mean and standard deviations were then calculated for each treatment.

For MA237 and MA238 strains containing the *thp* expression cassette, the effluent medium was investigated for antifreeze activity with a self-made cooling finger as previously described [25]. Additionally, determination of THP concentration was attempted using Centrifugal Filter Units (with cut off above 3 kDa, Amicon^®^Ultra), lyophilisation, different precipitations (TCA, acetone), or dialysis. Lyophilized biomass samples were grinded using pestle and mortar, and mycelium dissolved with protein extraction buffer (3.3 ml 0.5 M Na_2_HPO_4_, 6.6 ml 0.5 M NaH_2_PO_4_, 0.2 ml 0.5 M EDTA, 20 μl 100 mM PMSF, 1 ml 10 % SDS, pH 7.0). This protein solution and the different concentrated effluent were investigated using Tricine-SDS-PAGEs (16 %) [40], and Western blot with rabbit anti-THP primary antibody [26]. HPLC-ESI-MS analysis was conducted using an Orbitrap XL hybrid mass spectrometer (Thermo Fisher Scientific) coupled to a HPLC system 1200 (Agilent Technologies) as described by Jungmann et al. [41]. For SDS-PAGE separated proteins, gel spots on the expected height were digested (Trypsin In-Gel Digestion Kit, Thermo Scientific).

## Additional file

10.1186/s12934-016-0543-2 Design of Northern and qRT-PCR probes.

## Abbreviations

*A. niger*: *Aspergillus niger*
AFP: antifungal protein from *Aspergillus giganteus*
bp: base pair
cDNA: complementary DNA
CM: complete medium
ct: cycle threshold
DNA: deoxyribonucleic acid
EDTA: ethylenediaminetetraacetic acid
FPLC: fast protein liquid chromatography
g_DW_/kg: gram dry weight per kilogram culture broth
HPLC–ESI–MS: high performance liquid chromatography–electrospray ionization**–**mass spectrometric
*hygR*: hygromycin resistance gene
LCPS/OD: luminescent counts per second per optical density
*gpdA*: glyceraldehyde-3-phosphate dehydrogenase from *A. nidulans*
MIC: minimal inhibitory concentration
*mluc*: modified luciferase
MM: minimal medium
mRNA: messenger ribonucleic acid
*Panafp*: promoter of a gene encoding an antifungal protein in *Aspergillus niger*
PCR: polymerase chain reaction
*PglaA*: promoter of a gene encoding glucoamylase in *Aspergillus niger*
*PhfbD*: promoter of gene encoding a putative hydrophobin in *Aspergillus niger*
PMSF: phenylmethylsulfonyl fluoride
*pyrG*: orotidine-5′-decarboxylase gene from *Aspergillus oryzae*
qRT-PCR: quantitative reverse transcriptase PCR
SDS-PAGE: sodium dodecyl sulfate polyacrylamide gel electrophoresis
*SSanafp* and *SShfbD*: signal sequence for secretion of the respective genes
TCA: trichloroacetic acid
THP: thermal hysteresis protein from *Choristoneura fumiferana* isoform 337
*TtrpC*: the terminator of tryptophan synthase of *Aspergillus nidulans*
µ: specific growth rate
