# Supplementary material for: Highly active promoters and native secretion signals for protein production during extremely low growth rates in Aspergillus niger
Source: Microb Cell Fact. 2016 Aug 20;15:145. doi: 10.1186/s12934-016-0543-2 (PMC4992228; doi:10.1186/s12934-016-0543-2)
Supplement: Supplementary file 1 — 10.1186/s12934-016-0543-2 Design of Northern and qRT-PCR probes. [file 12934_2016_543_MOESM1_ESM.docx]

**Additional materials**

**Additional file 1:** Design of Northern and qRT-PCR probes.

| Northern probe |  | Digestion | | product size [bp] |
| --- | --- | --- | --- | --- |
|  | *mluc* | pMA247 [41] with PmeI | | 1667 |
|  | *thp* | pEN2 with XbaI and MluI | | 780 |
|  |  | Forward primer | Reverse primer |  |
|  | *act* | atctcccgtgtcgacatgg | gcggtggacgatcgagg | 656 |
| qRT-PCR probe | *gpdA* | AGGGCACCATCGAGACCTAC | TGGGTGGTGAAGACACCAGT | 145 |
|  | *h2B* | CTCGAAACTTGCCGCTTACA | ACTTCGTGACAGCCTTGGTG | 131 |
|  | *afp* | TGGCAAATGCTACAAGAAGG | AGCAGTAGCACTTCCCCTTG | 150 |
